# Comparison of the effects of three different (-)-hydroxycitric acid preparations on food intake in rats

**DOI:** 10.1186/1743-7075-2-23

**Published:** 2005-09-13

**Authors:** Johanna Louter-van de Haar, Peter Y Wielinga, Anton JW Scheurink, Arie G Nieuwenhuizen

**Affiliations:** 1 Department of BioMedical Research, Numico Research, PO Box 7005, 6700 CA Wageningen, the Netherlands; 2 Department of Neuroendocrinology, University of Groningen, PO Box 14, 9740 AA Haren, the Netherlands

## Abstract

**Background:**

Studies on the effects of (-)-hydroxycitric acid (HCA) in humans are controversial. As differences in the HCA preparations may contribute to this apparent discrepancy, the aim of the current study is to compare different HCA-containing preparations in adult Wistar rats.

**Design:**

The effects of 3 different HCA-containing preparations (Regulator, Citrin K, Super CitriMax HCA-600-SXS, all used at an effective HCA dose of 150 and 300 mg/kg, administered intragastrically) on food intake and body weight were studied in adult male Wistar rats. The efficacy was tested under 2 different experimental conditions: 1) after a single dose administration and 2) during repeated administration for 4 subsequent days.

**Results:**

Regulator and Citrin K significantly reduced food intake in both experimental setups, while Super CitriMax HCA-600-SXS was less effective. When administered for 4 subsequent days Regulator and Citrin K diminished body weight gain.

**Conclusion:**

Regulator and Citrin K were shown to be potent inhibitors of food intake in rats, whereas Super CitriMax HCA-600-SXS showed only small and more inconsistent effects. The striking differences in efficacy between these 3 preparations indicate that low doses of a relatively low-effective HCA preparation may have contributed to the lack of efficacy as found in several human studies.

## Background

(-)-Hydroxycitric acid (HCA) is widely used as an ingredient for nutritional supplements aimed at reduction of food intake, appetite and body weight. However, studies on the effects of HCA in humans are controversial. Four placebo-controlled studies support the efficacy of HCA in man. In these studies, HCA administration led to increased loss of body weight and appetite reduction [1,2], decreased energy intake [3] and increased fat oxidation [4]. In addition, one placebo-controlled study reported increased loss of body weight after combined treatment of HCA and chromium [5]. In contrast, several other studies have not confirmed these proposed effects of HCA on gain of body weight [6-10], energy intake [8-10] or substrate utilization [11,12] in man.

Several factors may contribute to these inconclusive results of the human studies on the efficacy of HCA. First, the doses used in the human studies are highly variable, typically ranging from 5 – 40 mg/kg HCA per day whereas in one trial a dose as high as 250 mg/kg was used [12]. Second, differences in HCA preparations or production processes may also contribute to above-mentioned inconsistency in the results. For instance, HCA may occur either in open chain or in a lactone form. Since the lactone form has shown to be a very less effective inhibitor of the citrate cleavage enzyme [13], different preparations attempt to prevent cyclization of HCA into its (ineffective) lactone by using different counter-ions (such as sodium, calcium or potassium).

To obtain some insight into the difference in efficacy of commercially available HCA preparations, we studied the effects of three different HCA preparations on voluntary food intake and body weight in conscious rats. The product names of these preparations were: Regulator, Citrin K and Super CitriMax HCA-600-SXS (abbreviated as CitriMax), respectively.

## Methods

### Animals and housing

All experimental protocols were approved by the Animal Experiments Ethical Committee DEC-Consult, Bilthoven, the Netherlands. Male Wistar rats (HsdCpb:WU, Harlan, the Netherlands) aged 3 months and weighing 290–320 gram at arrival were used. The rats were kept at 20 ± 1°C, with lights on from 23.00 h (ZT 0.00) until 11.00 h (ZT 12.00), and with water and RMH-B standard lab chow, containing (w/w) 24% protein, 52% carbohydrates and 6% fat (Hope Farms, Woerden, the Netherlands) *ad libitum *unless mentioned otherwise.

The rats received a permanent silicone cannula (I.D. 0.6 mm, O.D. 1.2 mm) in the stomach under Isoflurane/oxygen/nitrogen oxide anesthesia according to the method described by Strubbe et al. [14]. This was done to allow stress-free intragastric (ig) administration of components to freely moving rats. The animals were allowed to recover for at least one week after surgery.

### (-)Hydroxycitric acid preparations

The following preparations were used: (1) Regulator, a synthetic produced product, which contains 97% of a tri-potassium salt of HCA (HOB Ireland Limited, Dublin, Ireland), (2) Citrin K, an extract of *Garcinia cambogia*, which contains 50% HCA (Sabinsa Corporation, New Jersey, USA), with potassium as its primary mineral (28 g/100 g) and (3) Super CitriMax HCA-600-SXS (abbreviated as CitriMax), an extract of *Garcinia cambogia*, which contains 60% HCA (Interhealth Nutraceuticals Incorporated, Concord, California), containing K^+ ^(15 g/100 g) and Ca^2+ ^(11 g/100 g). To test whether the effects are specific to HCA, its structural analogue (4) tri-potassium citrate (Merck Eurolab B.V., Darmstadt, Germany) was used for comparison.

At a concentration of 75 mg HCA/ml demineralized water, the osmotic values of all preparations were 0.545 mOsm/l for Regulator, 0.507 mOsm/l for Citrin K, 0.265 mOsm/l for CitriMax and 0.490 mOsm/l for an equimolar solution of tri-potassium citrate in demineralized water.

### Experimental design

Two types of experiments were performed to study the potential differences in efficacy between the different HCA preparations. The first series of experiments focused on the effect of one single administration of each preparation on food and water intake for the following 46 hrs in a 4 days placebo-controlled crossover experiment. In the second series of experiments, HCA was given twice a day for 4 days to study effects of repeated doses of the component on food and water intake. In both studies, rats were housed individually in cages in which food and water intake was monitored online (UgoBasile, Comerio, Italy).

### Single administration

4 groups of 6 animals were used; within each group 2 single experiments were done. Each single experiment lasted for 4 days (96 hours). On day 1, food was removed 2 hours before dark onset (ZT 10.00). At ZT 11.30 randomly assigned rats received either a single bolus of the test component (dissolved in a total volume of 1 ml water) or a single bolus of isovolumic amount of water alone through the gastric cannula. At ZT 12.00 food was returned, and food and drink intake were monitored at 1, 2, 3, 4, 5, 6, 12, 24 and 46 hrs after administration. This period of 46 hrs was a washout period at the same time. On day 3 the protocol of day 1 was repeated. The rats that received the component on day 1 now receive vehicle and vice versa. Body weight was registered daily. In this way each dose of each HCA preparation was tested with regard to it's own vehicle treatment in a crossover experiment.

Each HCA preparation was tested, in a group of 6 animals, at two doses corresponding to 150 and 300 mg HCA/kg body weight. Thus, three groups of 6 animals were used to test Regulator at doses of 155 and 310 mg/kg, Citrin K at 300 and 600 mg/kg and CitriMax at 250 and 500 mg/kg, respectively. In the fourth group of 6 animals tri-potassium citrate, first, was tested at a dose of 475 mg per kg body weight (corresponding to the molarity of 300 mg/kg HCA) in comparison with vehicle treatment. Second, tri-potassium citrate (475 mg/kg) was tested in comparison with Regulator (310 mg/kg).

### Repeated administration

To study the effects of long-term administration of the different HCA preparations, HCA or vehicle was administered intragastric twice daily for 4 subsequent days (at ZT 11.30 and ZT 17.00). Body weight and food intake were monitored during the 4 days of ig HCA-administration and the 3 days thereafter at ZT 10.00.

For logistic reasons, the long term studies were performed in two subsequent series of experiments. In the first set of experiments (n = 15, for each treatment n = 5) vehicle, Regulator and CitriMax and in the second set (n = 27, for each treatment n = 9) vehicle, Regulator and Citrin K were tested. All tested doses corresponded to the dose of 300 mg HCA/kg body weight.

### Data analysis

In the single administration experiments, food intake were analyzed for the crossover experiments by a four-way ANOVA test with the factors, group of treatment order, animals within group, period and type of vehicle treatment, all per HCA preparation. First, the difference between time period and beginning was analyzed to test the possibility of a carryover effect. Because of the absence of a carryover effect, the type of vehicle treatment effects can be tested in the crossover experiment.

To compare the food intake results of the different HCA preparations in the single administration experiments, the delta (= difference) between treatment and vehicle on t = 24 hrs was calculated. To make comparisons between the HCA preparations, these deltas were analyzed using a two independent sample t-test with equal variances.

In the single administration experiments, bodyweight and water intake were also analyzed for the crossover experiments by a four-way ANOVA test, per HCA preparation.

In the repeated-administration experiments, the effects of HCA treatment on food intake, water intake and body weight were analyzed by a one-way ANOVA test. P values less than 0.05 were regarded significant. Data are presented as mean ± SEM.

## Results

### Single administration

**Figure 1:**
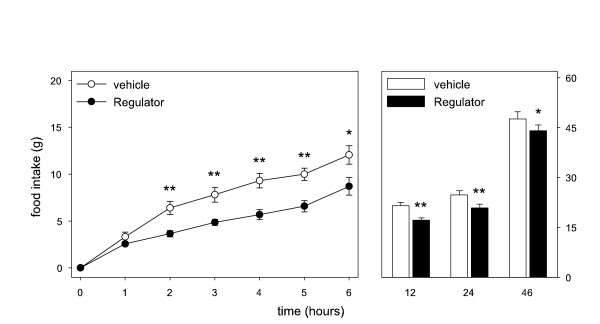
Cumulative food intake in rats up to 46 hrs after a single intragastric administration of vehicle or 310 mg/kg Regulator (n = 6). * p < 0.05, **p < 0.01

**Figure 2:**
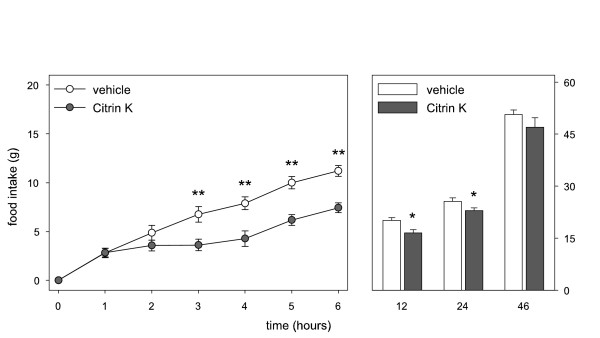
Cumulative food intake in rats up to 46 hrs after a single intragastric administration of vehicle or 600 mg/kg Citrin K (n = 6). * p < 0.05, **p < 0.01

**Figure 3:**
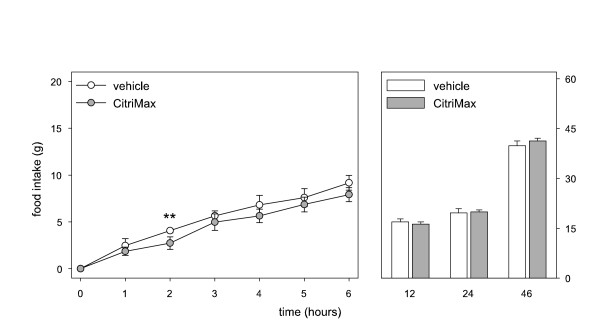
Cumulative food intake in rats up to 46 hrs after a single intragastric administration of vehicle or 500 mg/kg Super CitriMax HCA-600-SXS (n = 6). **p < 0.01

Cumulative food intake was significantly reduced after intragastric administration of 310 mg/kg Regulator (figure 1) and 600 mg/kg Citrin K (figure 2). CitriMax (500 mg/kg) tended to decrease food intake (figure 3), reaching significance at t = 2 h. The low doses of Citrin K (300 mg/kg) and Regulator (155 mg/kg) showed no effect on food intake, CitriMax (250 mg/kg) reduced food intake only at t = 2 h (table 1).

**Table 1:** Cumulative food intake in rats after a single intragastric administration of vehicle or different HCA preparations

		Regulator (155 mg/kg)		Citrin K (300 mg/kg)		Super CitriMax HCA-600-SXS (250 mg/kg)	
		Vehicle	Regulator	Vehicle	Citrin K	Vehicle	CitriMax
		
A	t = 1 h	2.9 ± 0.6	2.9 ± 0.4	2.9 ± 0.7	2.5 ± 0.6	2.9 ± 0.6	2.7 ± 0.4
	t = 2 h	5.7 ± 0.4	4.6 ± 0.5	4.2 ± 0.6	3.5 ± 0.6	4.3 ± 0.6	3.6 ± 0.7*
	t = 3 h	6.9 ± 0.6	6.0 ± 0.2	6.0 ± 0.9	5.0 ± 0.8	6.2 ± 0.5	5.2 ± 0.6
	t = 6 h	10.4 ± 0.8	10.4 ± 0.5	9.7 ± 0.9	9.3 ± 0.4	9.3 ± 0.7	9.2 ± 0.6
	t = 24 h	22.6 ± 0.8	23.6 ± 0.7	23.8 ± 0.7	23.0 ± 1.1	21.1 ± 1.2	21.5 ± 0.7
		Regulator (310 mg/kg)		Citrin K (600 mg/kg)		Super CitriMax HCA-600-SXS (500 mg/kg)	
		Vehicle	Regulator	Vehicle	Citrin K	Vehicle	CitriMax
		
B	t = 1 h	3.3 ± 0.5	2.5 ± 0.1	2.8 ± 0.5	2.8 ± 0.4	2.5 ± 0.7	1.9 ± 0.4
	t = 2 h	6.4 ± 0.7	3.6 ± 0.3**	4.9 ± 0.8	3.6 ± 0.6	4.1 ± 0.2	2.7 ± 0.7**
	t = 3 h	7.8 ± 0.8	4.9 ± 0.3**	6.8 ± 0.8	3.6 ± 0.6**	5.6 ± 0.5	5.0 ± 0.9
	t = 6 h	12.1 ± 1.0	8.7 ± 0.9*	11.2 ± 0.6	7.4 ± 0.5**	9.2 ± 0.8	7.9 ± 0.8
	t = 24 h	24.8 ± 1.2	20.9 ± 1.1**	25.6 ± 1.0	23.0 ± 0.8*	19.6 ± 1.2	20.0 ± 0.6

For each HCA preparation n = 6, * p < 0.05, ** p < 0.01

To enable comparison between the effects of the different HCA treatments, the difference between 24 hrs cumulative food intake after component treatment and after vehicle treatment was calculated for each HCA source (defined as delta, data shown in table 2). The negative delta's for Regulator (310 mg/kg) and Citrin K (600 mg/kg), implying a decreased food intake after HCA administration when compared to vehicle, were significantly different from the positive delta for CitriMax (500 mg/kg).

**Table 2:** Effect of intragastric administration of different HCA preparations on cumulative food intake in rats after 24 hrs.

Regulator	Citrin K	Super CitriMax HCA-600-SXS
(155 mg/kg)	(300 mg/kg)	(250 mg/kg)
0.6 ± 0.6	-0.8 ± 1.0	0.4 ± 1.1
Regulator	Citrin K	Super CitriMax HCA-600-SXS
(310 mg/kg)	(600 mg/kg)	(500 mg/kg)
-3.9 ± 0.6*	-2.7 ± 0.7*	0.4 ± 1.1

Data is calculated, for each HCA treatment, as the delta (= difference) between food intake after HCA treatment minus food intake after vehicle treatment. For each HCA preparation n = 6, * p < 0.05 in comparison with Super CitriMax HCA-600-SXS

**Figure 4:**
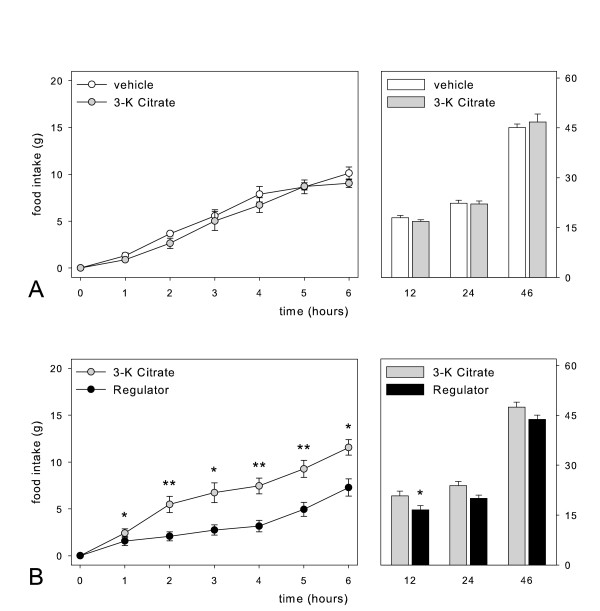
Cumulative food intake in rats up to 46 hrs after a single intragastric administration of A. vehicle or 475 mg/kg tri-potassium citrate (n = 6). B. 475 mg/kg tri-potassium citrate or 310 mg/kg Regulator (n = 6). * p < 0.05, **p < 0.01

Figure 4a shows that 475 mg/kg tri-potassium citrate had no effect on cumulative food intake compared to vehicle treatment. Regulator (310 mg/kg) decreased cumulative food intake compared to tri-potassium citrate (figure 4b).

No effect on body weight and water intake was observed in any of the experiments (data not shown).

### Repeated administration

**Figure 5:**
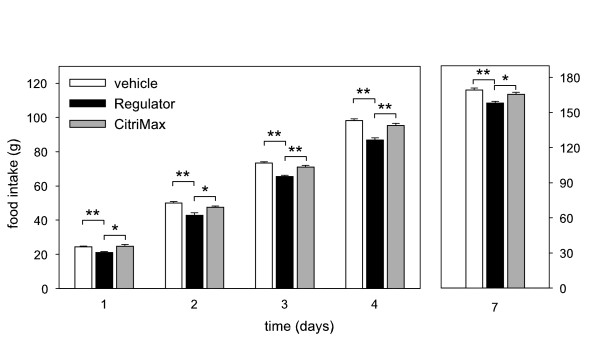
Cumulative food intake in rats during intragastric treatment twice daily for 4 subsequent days (day 1–4) and day 3 thereafter (day 7) with vehicle, 310 mg/kg Regulator or 500 mg/kg Super CitriMax HCA-600-SXS (for each treatment n = 5). * p < 0.05, **p < 0.01

**Figure 6:**
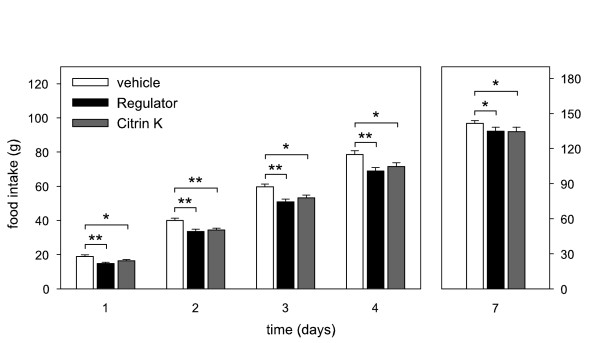
Cumulative food intake in rats during intragastric treatment twice daily for 4 subsequent days (day 1–4) and day 3 thereafter (day 7) with vehicle, 310 mg/kg Regulator or 600 mg/kg Citrin K (for each treatment n = 9). * p < 0.05, **p < 0.01

The effects of repeated administration of the high doses of the three preparations on food intake were comparable to the data of the single bolus experiments. Both Regulator and Citrin K significantly reduced cumulative food intake at days 1–4, whereas CitriMax did not reduce food intake (figure 5 and 6). Three days after the last administration, food intake was still significantly lower after Regulator and Citrin K treatment (figure 5 and 6). Figure 5 also shows that the cumulative food intake during and after Regulator treatment was significantly lower than during and after CitriMax treatment.

Regulator resulted in a significantly lower gain of body weight compared to vehicle at day 2 and 4. Citrin K significantly reduced gain of body weight compared to vehicle at day 1, 2 and 4 (data not shown).

## Discussion

This study shows that three commercially available HCA preparations exert striking differences in efficacy in inhibiting voluntary food intake in rats. Regulator and Citrin K were potent suppressants of food intake, both after a single bolus and after repeated administration, whereas CitriMax exerted much smaller effects on food intake. Accordingly, repeated administration of Regulator and Citrin K, but not CitriMax, reduced gain of body weight. Many of the peer-reviewed human studies on HCA, which report a lack of efficacy, used a CitriMax preparation as their source [9,10,12] at doses that were considerably lower than used in the present animal study, even when corrected for differences in metabolic rate. High doses of (CitriMax) HCA, however, have been shown to be effective in humans [2]. Therefore, the current study indicates that low doses of a relatively low-effective HCA preparation may have contributed to the lack of efficacy in several human studies.

The results of the current study are in line with earlier observations that HCA reduces food intake in rats [15-22]. Most of the early rat studies had not been carried out with the above-mentioned preparations; instead, a tri-sodium salt of HCA was used [15-18]. To our knowledge, no published rat studies used Regulator or Citrin K, while only the recent studies of Leonhardt et al. have been carried out with Super CitriMax HCA-600-SXG [19,20,22]. In these studies, Super CitriMax-600-SXG significantly inhibited food intake in rats, in contrast to the results obtained in our present studies. Differences in the experimental setup may underlie this apparent discrepancy. In their studies a different rat strain and a different experimental setup (10 days supplementation after substantial, fasting-induced body weight loss) were used. Also, a different CitriMax preparation (SXG instead of SXS) was used at a dose that was considerably higher than the maximal dose used in our single-dose administration studies (around 1000 mg/kg versus 500 mg/kg).

The cause of the differences in efficacy between the various HCA preparations remains speculative. In this study, it cannot simply be explained by differences in the relative HCA content of the used preparations. Regulator (97% pure for HCA) was equally effective as Citrin K (50% pure for HCA). Citrin K was more effective than CitriMax (60% pure for HCA). It cannot be excluded that the 40% non-HCA content of CitriMax contains component(s) that interfered with the suppressive effect of HCA on food intake. Alternatively, the extraction method may have resulted in an increased formation of (-)-HCA lactones, which are less potent inhibitors of the citrate cleavage enzyme [13].

It has been suggested that minerals play an important role in regulating the stability, bio-availability or solubility of HCA. In contrast to the two other more effective HCA sources, CitriMax has relatively a high calcium and relatively a low potassium content. Regulator and Citrin K contain negligible amounts of calcium and a relatively high content of potassium. If, as has been suggested, calcium reduces solubility and hinders bio-availability [23,24], the high calcium content in CitriMax may have negatively affected bio-availability or stability of the HCA molecule, resulting in a lower efficacy. It is unlikely that the high potassium content in the two other HCA sources is directly responsible for the observed inhibition of food intake, as tri-potassium citrate did not affect food intake.

The osmolarity of the CitriMax solution was considerably lower than the osmolarity of the more effective Regulator and Citrin K solutions. It has been suggested that osmolarity plays an important role in the regulation of satiety. Ingestion of hypertonic solutions decreased subsequent food intake in pigs [25]. In humans, the non-absorbable fructose stereoisomer D-tagatose inhibited food intake which may have been caused by the osmotic effects of the unabsorbed D-tagatose [26]. However, as the osmolarity of the ineffective tri-potassium citrate was as high as the osmolarity of Regulator and Citrin K, differences in osmolarity cannot solely account for the differences in efficacy of the various preparations used in this study.

It should be noted, however, that a decrease in food intake as a result of induction of malaise or discomfort by any of the used HCA sources cannot be excluded. Still, previous studies on the occurrence of conditioned taste aversion after HCA administration concluded that the food intake reducing effects of HCA cannot be solely explained by the induction of discomfort [27]. Accordingly, no behavioral signs indicating lack of well-being were recognized during these experiments.

In summary, this study shows that different commercially available HCA preparations show differences in their potency: Regulator and Citrin K are potent inhibitors of food intake in rats, whereas similar doses of CitriMax hardly showed any effect on food intake. As most of the (ineffective) human studies used low dose CitriMax preparations, the present study may indicate that low doses of a relatively low-effective HCA preparation may have contributed to the lack of efficacy in several human studies.

## Competing interests

The author(s) declare that they have no competing interests.

## Authors' contributions

JL: has made substantial contributions to conception and design, acquisition, analysis and interpretation of data and drafted the manuscript.

PW: has made substantial contributions to conception and design, acquisition, analysis and interpretation of data and has been involved in revising the article critically.

AS: has made substantial contributions to conception and design and has been involved in revising the article critically.

AN: has made substantial contributions to conception and design, has been involved in drafting the article and gave final approval of the version to be published.

All authors read and approved the final manuscript.
